# Epi-Mucosa Fixation and Autologous Platelet-Rich Fibrin Treatment in Medication-Related Osteonecrosis of the Jaw

**DOI:** 10.3390/dj9050050

**Published:** 2021-04-30

**Authors:** Antonio Cortese, Antonio Casarella, Candace M. Howard, Pier Paolo Claudio

**Affiliations:** 1 Unit of Maxillofacial Surgery, Department of Medicine and Surgery, University of Salerno, 84084 Fisciano, Italy; a.casarella1@studenti.unisa.it; 2 Department of Radiology, University of Mississippi Medical Center, Jackson, MS 39216, USA; chowardclaudio@umc.edu; 3 Department of Biomolecular Sciences, Maxillofacial Surgery, University of Mississippi, Jackson, MS 39216, USA

**Keywords:** medication-related osteonecrosis of the jaw, fracture, mandible, osteonecrosis, bisphosphonates

## Abstract

Medication-related osteonecrosis of the jaw (MRONJ) frequently affects patients after treatments with bisphosphonates or denosumab, especially with high doses in patients with bone osteoporosis, neoplastic metastases, or possibly anti-angiogenic treatment for cancer. The aim of this article was to show a new treatment planning for stage 2 and stage 3 MRONJ using platelet-rich fibrin (PRF) at the surgical field to enhance healing in association with a new epi-mucosal fixation technique to prevent or treat mandibular fracture. Two cases were treated by epi-mucosa fixation and autologous PRF use for prevention of mandibular fracture risks related to necrotic bone resection or a narrow fracture reduction. Both cases were successfully treated by this new technique of epi-mucosa fixation combined with autologous PRF and achieved good results and good quality of life. Ability to wear prosthesis with good mastication in the absence of side effect such as infection, plate and screw mobilization, pain, and other disabilities or extension of necrosis was reported. After surgical removal of necrotic bone, no infection was detected without any extension of the necrosis.

## 1. Introduction

Medication-related osteonecrosis of the jaw (MRONJ) is an adverse effect of treatment with denosumab or bisphosphonates (especially with high doses to prevent skeletal events in patients with bone and neoplastic metastases) or possibly anti-angiogenic treatment for cancer. Preventive measures in recent years have reduced the risk of MRONJ in patients with bone metastases due to cancer, particularly by avoiding trauma or invasive therapies and preventing or treating dental infections before and during therapy with denosumab or bisphosphonate. If MRONJ is developed, conservative medical treatment (non-surgical) can provide improvement, but the achievement of mucosal closure remains challenging. Symptoms management and mucosal healing are the ultimate goals of therapy but, after the failure of conservative treatment, a surgical approach may be beneficial. Overall, a multidisciplinary and pragmatic approach to MRONJ should be adopted, giving priority to quality of life and management of the patient’s malignant skeletal disease.

The stage of MRONJ at presentation is prognostic for the success of conservative (non-surgical) treatment, with only a very low likelihood of healing in patients who have higher stages of the disease [1]. Notwithstanding the disputes in the classification, we can distinguish three stages in the classification of MRONJ:

- Stage 1—In stage 1 the upper or lower jaw bone is exposed in the absence of symptoms; in this stage the treatment is conservative: Improve oral hygiene by actively treating dental and periodontal diseases; rinses with topical antibiotic. Surgical treatment is considered only to remove necrotic bone.
- Stage 2—In this stage we have exposed bone associated with pain and inflammation, swelling, or infection of adjacent soft-tissue. Treatment is like that in stage 1 with the addition of systemic antibiotic to treat any infection and symptomatic therapy.
- Stage 3—In the third stage we have same staging criteria of stage 2 plus at least one of these: Pathological fracture, extra-oral or oroantral fistula and radiographic evidence of osteolysis extending to inferior border of mandible or floor of maxillary sinus. Treatment is like that in stage 2 with administration of systemic antibiotics. However, in extended cases we consider a surgical resection of the jaw and reconstruction.

Although osteonecrosis of the jaw (ONJ) is typically diagnosed clinically, the use of orthopantomography, CT, and/or MRI are necessary. Another problem that should be considered in the staging of MRONJ is the lack of dimensional criteria; currently the disease affecting an entire quadrant can be staged in the same way as a lesion with a diameter of less than 1 cm, even if the probability of favorable outcome can be totally different.

Clinical observation has shown an association between the onset of MRONJ and dental extractions and infection, although the underlying mechanism of how these events lead to osteonecrosis remains poorly understood, and high-quality evidence is required. Other factors that negatively influence MRONJ have been reported in the literature as being associated with its accelerated development and an increase in the severity of the condition, but their causal association is not clear [1,2]. These negative factors include:

- Use of corticosteroids;
- Presence of diseases or concomitant conditions (e.g., pre-existing dental infections, anemia, diabetes mellitus and immunosuppression, or renal failure);
- Poor oral hygiene and smoking [1].

AAOMS emphasizes that priority should be given to cancer treatment for patients with cancer and bone metastases at risk of developing MRONJ [1]. We need to balance the risk of MRONJ with the benefit of bisphosphonates or denosumab in reducing the substantial risk of SREs [1].

A combination of preventive measures taken both before and during treatment with denosumab or bisphosphonates can significantly reduce the risk of MRONJ1 [1,2].

The aim of the work is to show new procedure and new planning for second-third stadium (heavy stadium) for medication-related osteonecrosis of the jaw (MRONJ), which is most conservative as possible and also effective in preventing mandibular fracture in risky cases or in a fractured affected case. In these cases, because of published evidence that osteonecrosis of the jaw is related to surgical aggressive therapies, there is a contradiction between etiology of the MRONJ and the most effective treatment with surgical procedure. To solve this problem, we developed this new therapeutic planning using an epi-mucosal fixation technique from our experience in orthognathic and orthopedic maxillofacial surgery. In this way, close reduction of the fracture and close stabilization of the fracture was achieved, minimizing surgical approach and surgical effect impact on the affected bone area. Stabilization of the affected bone segment can be achieved by fixing the plate using self-locking screws in the affected bone segment through the epi-mucosal technique without any need for periosteal elevation or mucosa incision. Autologous platelet-rich fibrin (PRF) was used, combined with fixation to improve the healing abilities of the bone segment and the mucosa after bone sequestrum removal and shaving of the sharp bone borders of the affected area.

Many studies have shown that PRF is a healing biomaterial with great potential. PRF stimulates bone and soft tissue regeneration, without inflammatory reactions, which can be used alone or in combination with bone grafts, promoting hemostasis, bone growth, and maturation [3,4]. PRF consists of a fibrin matrix rich in platelets and autologous leukocytes with a tetra-molecular structure, with cytokines, platelets, and stem cells inside. Therefore, it acts as a biodegradable scaffold improving micro-vascularization and is also able to guide the epithelium in cellular migration on the surface [3,4]. The routine use of such an inexpensive and affordable autologous grow factor delivery system certainly offers an interesting option for the treatment of horizontal defects [3,4,5,6].

The aim of this study was to develop a protocol for the treatment of stage 2 and stage 3 MRONJ with extensive bone necrosis causing segment fracture or high-risks for fracture. We combined the advantages of using PRF with the advantages of surgical removal of the necrotic bone and stabilization of the pathologic fracture of the necrotic bone segment that prevents fracture risks of the affected segments after resections or sequestrectomy. To avoid periosteum elevation during rigid bone fixation, we adopted a new technique for rigid fixation: The epi-mucosal fixation. This technique was already validated and published for jaws reducible fractures: It was the first time it was adopted in MRONJ cases, hence the scientific relevance. The study follows the rules of our Ethical Committee.

## 2. Materials and Methods

Two cases are described in this study.

Both cases were examined by X ray panoramic and CT scan pre-operatively and in the short post operatively time. 

The first case was a 72-years-old woman of Caucasian origin, who was referred to the maxillo-facial unit of the University Hospital of Salerno complaining of pain in the region of the left side of the mandible. The patient had received a mastectomy in 2013 in another hospital for a primitive breast cancer. Since 2014 she was treated with denosumab for bone metastases secondary to a primitive cancer.

**Figure 1:**
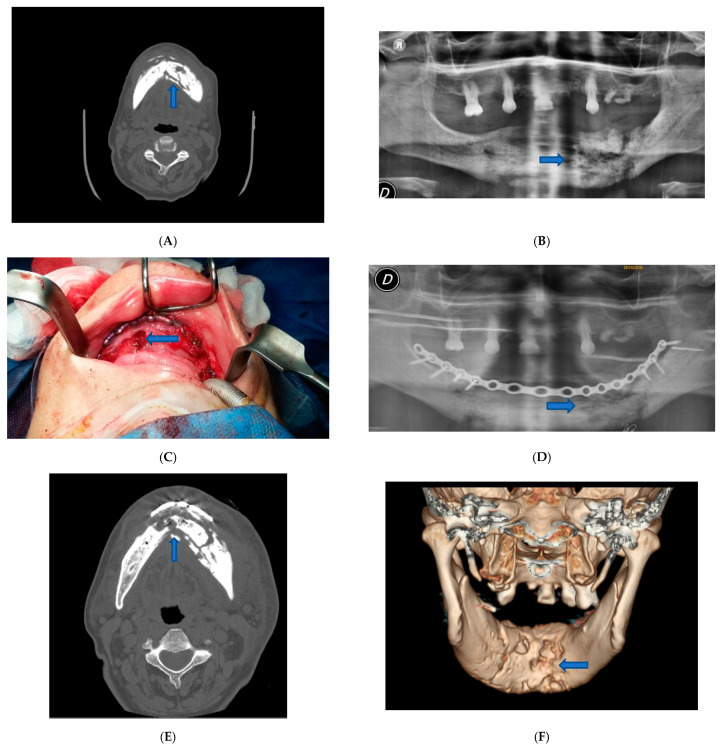
Patient 1. (**A**) Pre-operative CT scan image showed lithic area in the mandibular horizontal branches, which appeared subverted and fractured on the left side. (**B**) Pre-operative panoramic X-ray image of the dental branch revealed lithic area. (**C**) Epi-mucosal fixation performed using SMART Lock screws and plates without elevating the mucoperiosteal flap. (**D**) Post-operative panoramic X-ray image of the dental branch. (**E**) Post-operative CT scan image. (**F**) Post-operative CT scan 3D reconstruction.

Surgery was performed under general anesthesia. We proceeded to the superficial removal of damaged tissues in the site of the osteonecrosis outbreak to avoid extension of the necrosis and to enhance healing abilities. We eliminated all the necrotic tissue, saving healthy tissue at the excision border to preserve as much as possible mandibular contour aesthetic and function. To stabilize the bone segment, an epi-mucosal fixation was performed using SMART Lock screws and plates without elevation of the mucoperiosteal flap, therefore avoiding any new bone necrosis risk (Figure 1C).

The self-locking screws and plate were inserted before the necrotic bone area resection or sequestrectomy by epi-mucosa plate modelling, positioning and fixing with trans mucosa screw insertion. No need for mucosa incision or periosteal elevation was required at this step of the procedure; the self-locking screws and plate acted as a rigid external fixation system.

Next, we proceeded to the preparation of autologous PRF to be used as a regenerating and healing stimulating material by collecting 40 mL of peripheral blood from the patient at the time of surgery. Blood samples were collected in 8.5 mL tubes without any anticoagulant and immediately centrifuged at 2700 rpm for 12 min to prevent activation of the coagulation cascades. A model Hettich^®^ (Westphalia, Germany) EBA 20 of centrifuge with a fixed eight-point angular rotor was used for the procedure. Centrifugation time changed according to the consistency required for the PRF; the longer the centrifugation time, the harder the consistency of the PRF sample. After centrifugation, a PRF from the center of the tube was obtained; the centrifuged red blood cells at the bottom and the acellular plasma at the top were discarded. The PRF was inserted directly into the osteotomy slot after sequestrectomy and sharp bone trimming. Several studies have assessed the efficacy of PRF in intra-osseous and mandibular grade 2 MRONJ defects and found a positive clinical and radiographic outcome [7,8].

A vicryl polyglactin (91, 3/0) absorbable suture was used to close the flap.

The postoperative course was without complications. Postoperative therapy comprised oral hygiene instructions, rinsing with 0.2% chlorhexidine solutions twice a day, and an evening application of 0.2% chlorhexidine gel upon the sutured incision lines, as well as the administration of a non-steroidal anti-inflammatory aid (Ketoprofen 80 mg) for five consecutive days. The patient was also administered antibiotic therapy in the perioperative phase, starting the night before surgery for 4 days, using 500 mg of Amoxicillin and Clavulanate every 8 h. The patient was examined for the first time one week later and then 3 and 6 months after surgery (Figure 1D–F).

The second case was an 80-years-old woman of Caucasian origin who referred bilateral pain in the mandibular body. The patient was treated with continuous therapy with bisphosphonates.

**Figure 2:**
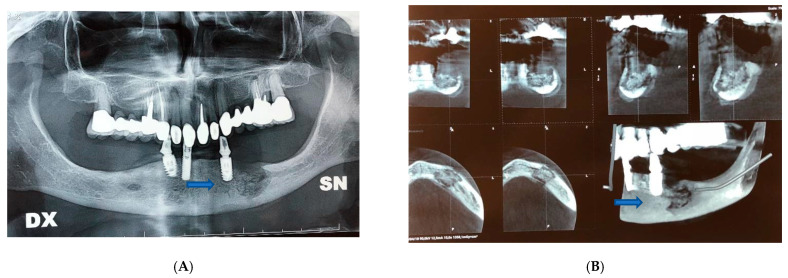

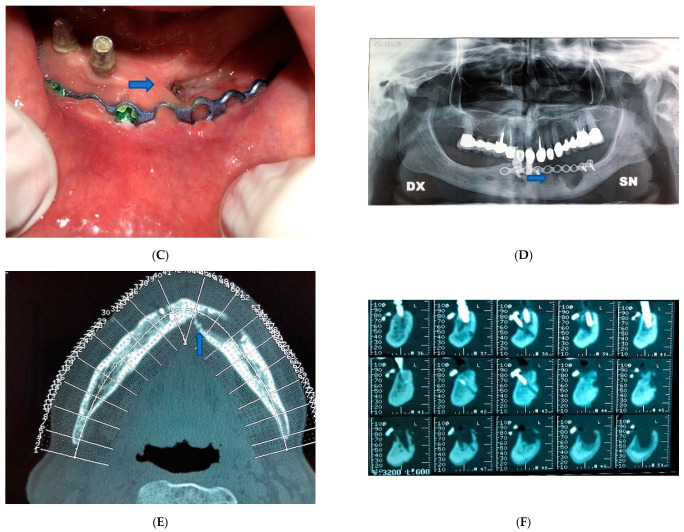
Patient 2. (**A**) Pre-operative panoramic X-ray examination showed no fractures, but there was a subversion of the normal organization of the bone. (**B**) Pre-operative X-Ray image showed lithic area in the lower branch. (**C**) Epi-mucosal fixation performed using SMART Lock screws and plates without elevating the mucoperiosteal flap. (**D**) Post-operative panoramic X-ray image of the dental branch revealed lithic area. (**E**) Post-operative CT scan Image. (**F**) Post-operative X-ray image.

In this second case, despite the absence of fractures, it was decided to intervene and apply an epi-mucosal fixation because of the very high risk of pathologic fracture due to the osteonecrosis outbreak. Surgery was performed under general anesthesia. After the debridement and removal of the necrotic bone with sequestrectomy and bone trimming, also in this case we proceeded to an epi-mucosal fixation performed using SMART Lock screws and plates without elevating the mucoperiosteal flap (Figure 2C). As in the first case, we proceeded to the preparation of autologous PRF with the application of the PRF directly to the site of intervention. A vicryl polyglactin (91, 3/0) absorbable suture was used to close the flap.

Postoperative course was without complications. Postoperative therapy comprised oral hygiene instructions. Application of 0.2% chlorhexidine solutions twice a day, an evening application of 0.2% chlorhexidine gel upon the sutured incision lines, and administration of a non-steroidal anti-inflammatory aid (Ketoprofen 80 mg) for five consecutive days. Even in this case, antibiotic therapy was administered in the perioperative phase, starting the night before the surgery and up to 4 days after, using 500 mg of Amoxicillin and Clavulanate every 8 h. The patient was examined for the first time one week later and then 3 and 6 months after surgery (Figure 2D–F).

Post-operative pain was assessed with the VAS score. The VAS score is a tool for measuring the subjective characteristics of pain experienced by the patient. The scale consists simply of a strip of 10 cm paper at the ends presenting two “end points” that are defined as “no pain” and the “worst pain that I can imagine”. The patient is asked to mark the pain at a point on the scale as perceived at that moment. The interval between the two ends (end points) is marked every centimeter and allows us to assign a value to the pain perceived by the patient. The initial score can be used as a subjective assessment of the pain experienced by the patient. The subsequent measurements required allow health professionals to understand if the pain is actually reducing and to what extent.

Ability to wear a prosthesis was possible also using the epi-mucosal fixation devices for stabilizing the dental prostheses, with the miniplates and self-locking screws acting as a bar supporting the prostheses. 

## 3. Results

For case one, patient physical examination showed no obvious swelling of the face. CT examination showed a lithic area in the mandibular horizontal branches, which appeared subverted and fractured on the left side (Figure 1A). A panoramic X-ray image of the dental arches has revealed the same characteristics (Figure 1B). The alteration was bilateral but particularly accentuated on the left. An additional bone lysis area was also appreciated at the D8 body level. A pathological fracture with radiographic evidence of osteolysis extending to the inferior border of the mandible was compatible with a diagnosis of stage 3 MRONJ caused by the continuous use of denosumab for three years (administration of 1 vial subcutaneous every six months). Additionally, fistulas were present at the lower border of the mandible.

For case two, patient physical examination showed no obvious swelling of the face. Oral examination showed no irregularities.

CT examination showed that there was a lithic area in the lower branch of the mandible. Although there were no fractures, the subversion of the normal organization of the bone exposed the patient to a high-risk for pathological fracture. Radiographic evidence showed an extensive osteolysis compatible with stage 3 MRONJ (Figure 2A,B). 

In both the presented cases treated by our protocol associating surgical removal of the necrotic bone outbreak by surgical drilling and smoothing of sharp edges in the affected segment followed by application of autologous PRF we achieved good results. Post-operative clinical conditions were evaluated by analyzing the following results:

- Mucosal integrity: Absence of necrotic bone exposure;
- Absence of residual infection: Absence of purulent exudate and reduction of swelling;
- Pain reduction: Evaluation of the VAS score.

We achieved good bone healing with absence of necrotic bone exposure, mucosa healing with complete necrotic site closure, and absence of residual infection without any swelling or residual fistulas.

Patients were able to wear a dental prosthesis without any referred pain at rest or during soft diet mastication.

We followed the first patient with periodic controls at one, three, and six months. In the post-operative controls, we could detect a progressive healing of the mucosa up to a final result, with complete mucosa healing without any signs of necrosis or infection. A significant reduction in pain was assessed by the VAS scale. Before the operation she reported a pain score on the VAS scale between 9 and 10, with the need to use painkillers. A few days after the operation, she had already reduced the use of painkillers. In the three-month post-operative control, she completely eliminated the use of painkillers, having a score on the VAS scale of 0, with no pain. The patient died two years after the operation as a result of breast cancer general complications.

In the second case, after the operation, we also followed the patient with periodic controls at one, three, and six months. In post-operative controls we observed that there was a progressive healing of mucosal integrity and infection. In this second case too, the patient had a progressive reduction of pain and no signs of necrosis or infection. Before the operation she reported a pain score on the VAS scale of about 8, with the need to use painkillers. Shortly after the operation she reduced the use of painkillers. In the one-month postoperative checkup she eliminated the use of painkillers, having a score on the VAS scale of zero with no pain.

## 4. Discussion

Both cases were treated with our new protocol, consisting of the combination of a well-demonstrated conservative treatment by autologous PRF and surgical removal of the necrotic bone area combined with epi mucosa mandibular fixation in case of fracture risks or conclamant fracture in the necrotic bone segments. By our new original epi-mucosal fixation technique it was possible to achieve close fixation of the segment, avoiding large periosteal elevation in the affected area of the jaws.

In the scientific literature, high doses of bisphosphonates or denosumab are associated with an increased risk of MRONJ compared to low dose regimens [1].

For patients who received continuous denosumab during the blinded treatment phase plus the open-label extension phase, the incidence of confirmed MRONJ, adjusted for years of patient follow-up, was 1.1% during the first year of denosumab treatment, 3.7% in the second year, and 4.6% per year thereafter. The median cumulative exposure to denosumab was 43.0 months for those patients with breast cancer (*n* = 318) and 36.9 months for those with prostate cancer (*n* = 147) [1].

Denosumab does not become physically bound to the bone matrix and consequently is associated with low levels of accumulation compared with bisphosphonates, which may remain covalently bound to the bone for many years due to its mode of action. The effects of denosumab are reversed faster on suspension of treatment [1].

Tooth extractions in patients receiving denosumab or bisphosphonate treatment, especially in the oncological setting, should be performed under antibiotic prophylaxis (e.g., amoxicillin/clavulanic acid) and accompanied by smoothening of sharp bony edges and closure of the wounds, and then monitored until complete mucosal healing is achieved.

In general, well-designed prospective clinical trials and non-interventional studies that investigate MRONJ management approaches in patients with cancer and bone metastases are lacking. Furthermore, inconsistent outcome measures (e.g., patient QoL scales, mucosal healing, and symptom improvement) are used in all studies [1]. This means that it is difficult to reach a consensus on the most effective MRONJ management approaches available. Patients who develop MRONJ should be referred to a maxillofacial surgeon or an oncologist [1]. A pragmatic approach to the management of patients with MRONJ should be adopted, with the management of malignant skeletal disease at the forefront of the care of each patient. Conservative management is recommended for the AAOMS I MRONJ stage, supplemented with appropriate surgical approaches for the most severe cases.

The current AAOMS guide recommends soft tissue debridement to treat irritation and infection in association with necrotic bone removal in stage 2, while bone resection at the necrotic area is considered in stage 3 [1]. The main problems related to surgery will consist of: temporary decrease in quality of life during convalescence, risks of relapse with extension of the necrosis with a second stage surgical need, possibility of complications (e.g., risk of infection or fracture), need for extended hospitalization, uncertain implications of interruptions necessary to chemotherapy and any cost per patient, and final treatment failure.

In our study, we developed a new protocol for the treatment of patients in stage 2 and stage 3 MRONJ causing segment fracture or showing high-risk for fracture. In MRONJ treatment there is a contraposition between two requirements: One is to remove the necrotic bone area, the other one is to prevent new necrosis by avoiding periosteal elevation in affected the area. To solve these two opposite requirements, we developed a new technique where bone resection was performed and fixation of the fractured bone segments in the affected area or preventive fixation of the resected area at risk of fracture was performed by our epi-mucosa fixation technique. This technique was already validated and published for jaw reducible fractures; because of the similar condition for reducible jaw fractures in MRONJ cases and in non-affected patients, we adopted this technique in the two reported cases. It was the first time the technique was adopted in MRONJ cases, hence the scientific relevance. In this way, it was possible to avoid periosteal elevation with a subsequent bone nutrition impairment and necrosis extension risk. In this new technique, the fixation is performed in closed surgery by fixing the bone plates over the mucosa using self-locking screws, as in an external fixation system. Conservative treatment by autologous PRF is also used in combination, to enhance and promote soft tissue and exposed bone area healing.

We developed the epi-mucosal fixation technique for reducible or well aligned fractures of the jaw; in these cases, fixation of the two bone segments is performed after close reduction of the fracture, avoiding surgical trauma and periosteal elevation, thereby preserving bone nutrition. In this way, we can ensure less surgical trauma with earlier healing of the lesion for the patient.

As shown in our previous study, the main indications for this technique were in well aligned fractures of the jaws (especially in elderly edentulous patients when minimally invasive techniques are needed) or in dislocated jaw fractures that can be reduced by closed manipulation without any incision. Particular indication for this technique is in case of multi segmented fractures with high risks for bone resorption and infection in case of periosteum elevation because of lack of nutrition of the bone fragments [9]. Long threaded-necked screws and plates are used, avoiding plate compression on the muco-periosteum and bone fragments, thus using a self-locking screws and plates system like external fixers. The bone fractures are fixed in position, after the reduction, by a rigid fixation system without compression that joins the insertion of the screw into the bone fragment on one side and the insertion of the thread screw neck in the holes of the plates on the other, obtaining a three-dimensional rigid system without any incision [8,10].

The main advantages of this technique are the minimum surgical trauma suitable for elderly patients, avoiding the risk of devascularization of the fragment even in comminuted or multifocal fractures; in this way, the quality and speed of the healing process is improved [9].

Other advantages are an easier removal of fixing devices, avoiding further surgical time, unlike in current techniques with sub-periosteal miniplates and screws, and no risk of periodontal damage, unlike in ferule methods, with lower costs and greater benefits of results [9,11]. It is conceivable to develop this technique and apply a double-plate fixation system for each fracture line in case greater stability is required, such as in unfavorable jaw line fractures, particularly in larger fractured areas [9].

With the use of this new epi-mucosa fixation technique, we were able to shorten the healing time with minimal surgical impact on the patients affected by MRONJ with fractured or weakened bone segments. In conclusion, the intraoral epi-mucosal fixation system is comparable to external fixation devices, with the advantages of avoiding trans-cutaneous pins or cumbersome devices with aesthetic and functional impairment. For the minimum device size, intraoral placement of the system is also possible for the molar areas using a ratchet screwdriver and contra-angle tips. Particular attention must be paid to the selection of the screw insertion sites in the interalveolar bone septa, avoiding damage to the dental roots.

## 5. Conclusions

Analyzing the result of the two cases treated by our technique, it was possible to achieve good results, combining the most effective treatment, even for advanced cases of MRONJ (stage 3). Excellent results were obtained, limiting risks and disadvantages, also in cases with high-risk for bone fracture or cases of effective fractured segment in necrotic bone segments. 

The advantages are: Non-invasive surgical procedure; ability to stabilize a bone segment at risk for pathologic fracture; limited risks for necrosis extension; ability in solving pain and necrosis related symptoms achieving mucosa healing.

In our cases, we did not find any complications related to pain during plate and screw fixation, and there was no infection around the sites of screw insertion and no screw weakening or plate mobilization. 

Even if the epi-mucosal fixation procedures are already used in close reducible maxillofacial fractures, the number of cases was limited, and additional studies with a larger number of cases are needed to finally assess the reliability and effectiveness of this procedure in MRONJ patients, even if showing promising results in our cases.

## Data Availability

Data is available upon reasonable request.

## References

[1] Otto S., Pautke C., Van den Wyngaert T., Niepel D., Schiødt M. (2018). Medication-related osteonecrosis of the jaw: Prevention, diagnosis and management in patients with cancer and bone metastases. Cancer Treat. Rev..

[2] Otto S. (2015). Medication-Related Osteonecrosis of the Jaws.

[3] Cortese A., Pantaleo G., Caggiano M., Amato M. (2016). Platelet-rich fibrin (PRF) in implant dentistry in combination with new bone regenerative technique in elderly patients. Int. J. Surg. Case Rep..

[4] Cortese A., Pantaleo G., Ferrara I., Vatrella A., Cozzolino I., Di Crescenzo V., Amato M. (2014). Bone and soft tissue non-Hodgkin lymphoma of the maxillofacial area: Report of two cases, literature review and new therapeutic strategies. Int. J. Surg..

[5] Dohan D.M., Del Corso M., Charrier J.B. (2007). Cytotoxicity analyses of Choukron’s platelet-rich-fibrin (PRF) on a wide range of human cells: The answer to a commercial controversy. Oral Surg. Oral Med. Oral Patholog. Oral Radiol. Endod..

[6] Kang Y.H., Seon S.H., Park J.H., Choung Y.H., Choung H.W., Kim E.S., Choung P.H. (2011). Platelet-rich fibrin is a Bioscaffold and reservoir of growth factor for tissue regeneration. Tissue Eng. Part A.

[7] Shin W.J., Kim C. (2018). Prognostic factors for outcome of surgical treatment in medication-related osteonecrosis of the jaw. J. Korean Assoc. Oral. Maxillofac. Surg..

[8] Cano-Duran J.A., Pena-Cardelles J.-F., Ortega-Concepcion D., Paredes-Rodriguez V.M., Garcia-Riart M., Lopez-Quiles J. (2017). The role of Leucocyte-rich and platelet-rich fibrin (L-PRF) in the treatment of the medication-related osteonecrosis of the jaws (MRONJ). J. Clin. Exp. Dent..

[9] Cortese A., Savastano G., Amato M., Pantaleo G., Claudio P.P. (2014). Intraoral epimucosal fixation for reducible maxillary fractures of the jaws; surgical considerations in comparison to current techniques. J. Craniofac. Surg..

[10] Cortese A., Savastano M., Cantone A., Claudio P.P. (2013). A new palatal distractor device for bodily movement of maxillary bones by rigid self-locking miniplates and screws system. J. Craniofac. Surg..

[11] Cortese A., Savastano M., Savastano G., Claudio P.P. (2011). One-step transversal palatal distraction and maxillary repositioning: Technical considerations, advantages, and long-term stability. J. Craniofac. Surg..

